# Genesis and Spread of Novel Highly Pathogenic Avian Influenza A(H5N1) Clade 2.3.4.4b Virus Genotype EA-2023-DG Reassortant, Western Europe

**DOI:** 10.3201/eid3106.241870

**Published:** 2025-06

**Authors:** Steven Van Borm, Ann Kathrin Ahrens, Claudia Bachofen, Ashley C. Banyard, Cathrine Arnason Bøe, François-Xavier Briand, Zuzana Dirbakova, Marc Engelsma, Alice Fusaro, Evelien Germeraad, Britt Gjerset, Béatrice Grasland, Frank Harders, Pierre Hostyn, Ari Kauppinen, Bénédicte Lambrecht, Benjamin C. Mollett, Isabella Monne, Alexander Nagy, Anne Pohlmann, Daniel Polzer, Scott M. Reid, Sandra Revilla-Fernandez, Mieke Steensels, Michaela Stätter, Edyta Swieton, Niina Tammiranta, Michele Wyler, Bianca Zecchin, Siamak Zohari, Simon Dellicour

**Affiliations:** Avian Virology and Immunology Unit, Sciensano, Brussels, Belgium (S. Van Borm, P. Hostyn, B. Lambrecht, M. Steensels); Institute of Diagnostic Virology, Friedrich Loeffler Institut, Greifswald-Insel Riems, Germany (A.K. Ahrens, A. Pohlmann); Institute of Virology and Immunology, Federal Department of Home Affairs, Mittelhäusern, Switzerland (C. Bachofen, M. Wyler); World Organisation for Animal Health/Food and Agriculture Organization of the United Nations International Reference Laboratory for Avian Influenza and Newcastle Disease, Animal and Plant Health Agency-Weybridge, Addlestone, UK (A.C. Banyard, B.C. Mollett, S.M. Reid); Norwegian Veterinary Institute, Ås, Norway (C. Arnason Bøe, B. Gjerset); Agence Nationale de Sécurité Sanitaire de l’alimentation, de l’environnement et du travail, Ploufragan-Plouzané-Niort, France (F.-X. Briand, B. Grasland); State Veterinary Institute Department of Animal Health, Zvolen, Slovakia (D. Dirbakova); Wageningen Bioveterinary Research Department of Virology, Lelystad, the Netherlands (M. Engelsma, E. Germeraad, F. Harders); European Reference Laboratory for Avian Influenza and Newcastle Disease, Istituto Zooprofilattico Sperimentale delle Venezie, Padua, Italy (A. Fusaro, I. Monne, B. Zecchin); Finnish Food Authority, Helsinki, Finland (A. Kauppinen, N. Tammiranta); Department of Molecular Biology, State Veterinary Institute Prague, Praha, Czech Republic (A. Nagy); Austrian Agency for Health and Food Safety, Institute for Veterinary Disease Control, Mödling, Austria (D. Polzer, S. Revilla-Fernandez, M. Stätter); National Veterinary Research Institute Department of Research Support, Puławy, Poland (E. Swieton); Swedish Veterinary Agency Department of Microbiology, Uppsala, Sweden (S. Zohari); Spatial Epidemiology Lab (SpELL), Université Libre de Bruxelles, Brussels, Belgium (S. Dellicour); Laboratory for Clinical and Epidemiological Virology, Department of Microbiology, Immunology and Transplantation, Rega Institute, KU Leuven, Leuven, Belgium (S. Dellicour); Interuniversity Institute of Bioinformatics in Brussels, Université Libre de Bruxelles, Vrije Universiteit Brussel, Brussels (S. Dellicour)

**Keywords:** Influenza, zoonoses, viruses, highly pathogenic avian influenza virus, HPAI, H5­N1, whole-genome sequencing, phylogeography, Europe

## Abstract

In Europe, highly pathogenic avian influenza (HPAI) virus circulates in avian wildlife, undergoing frequent reassortment, sporadic introductions in domestic birds, and spillover to mammals. An H5N1 clade 2.3.4.4b reassortant, EA-2023-DG, affecting wild and domestic birds was detected in western Europe in November 2023. Six of its RNA segments came from the EA-2021-AB genotype, but the polymerase basic 2 and polymerase acidic segments originated from low pathogenicity avian influenza viruses. Discrete phylogeographic analyses of concatenated genomes and single polymerase basic 2 and polymerase acidic segments suggested reassortment in summer 2023 near the southwestern Baltic Sea. Subsequent continuous phylogeographic analysis of all concatenated EA-2023-DG genomes highlighted circulation in northwestern Europe until June 2024 and long-distance dispersal toward France, Norway, England, Slovakia, Switzerland, and Austria. Those results illustrate the value of phylodynamic approaches to investigate emergence of novel avian influenza virus variants, trace their subsequent dispersal history, and provide vital clues for informing outbreak prevention and intervention policies.

Since 2016, Europe has experienced periodic introductions of clade 2.3.4.4b highly pathogenic avian influenza (HPAI) subtype H5 viruses via wild migratory bird movements and sporadic spillover events to poultry, which have resulted in several outbreaks affecting wildlife and poultry (*1*). In 2021, the typically seasonal epidemiologic cycle changed, and HPAI viruses became enzootic in Europe (*2*). The hemagglutinin (HA) gene of HPAI clade 2.3.4.4 viruses (*3*) showed rapid wild bird–mediated expansion, including global spread (*4*), and rapid evolution in all affected geographic areas (*5*–*7*). After 2021, increased HPAI incidence in Europe resulted in greater genetic diversity and in the evolution of new genotypes by frequent reassortment events. By 2022, the continued circulation of HPAI viruses led to numerous poultry outbreaks, affected an increasing number of wild bird species, and led to frequent reassortment, making assessment of the epidemiologic situation difficult (*2*). Sporadic infection of mammals is observed, including the unexpected spread to cattle in North America and cases of human infection (*8*,*9*), and is sometimes associated with adaptive mutations for viral replication in mammalian cells. Those observations stress the zoonotic risk associated with this particular HPAI virus (HPAIV) clade and the need for surveillance efforts, including whole-genome sequencing.

By the end of 2023, continued circulation and frequent reassortment events resulted in the co-circulation of 11 H5Nx HPAIV genotypes in Europe, 7 of which emerged during the fall of 2023 (*10*,*11*). One of those novel reassortants was assigned genotype EA-2023-DG, according to the standardized avian influenza virus (AIV) genotyping nomenclature in Europe (*2*). EA-2023-DG was first reported in Germany in November 2023 and shared most of its genomic segments with the contemporary dominating genotype EA-2021-AB but also gained polymerase basic protein (PB) 2 and polymerase acidic protein (PA) segments from low pathogenicity avian influenza viruses circulating in western Europe (*10*). Using all available EA-2023-DG genotype complete genome sequences, we conducted recombination and discrete as well as continuous phylogeographic analyses to investigate the emergence and subsequent dispersal dynamic of this genotype. 

## Methods

### Data Selection and Genotyping

The European Union Reference Laboratory, national reference laboratories, and some other partners perform sequencing analyses of the complete genome of HPAI H5Nx viruses detected through ongoing surveillance programs. All sequences are deposited in the GISAID EpiFlu database (http://www.gisaid.org). Partners perform genotyping on the basis of the phylogenetic tree topology, using previously described methods (*2*). During November 1, 2023–June 25, 2024, genotyping identified a total of 54 virus genomes as belonging to genotype EA-2023-DG. We extracted publicly available complete genomes comprising 8 segments from GISAID EpiFlu on January 15, 2025, resulting in a dataset consisting of all 54 identified EA-2023-DG genomes associated with exact temporal and spatial sampling metadata (Appendix 1 Table 1). The earliest publicly available EA-2023-DG genome, A/*Gallus_gallus*/Belgium/11307_0002/2023 (GISAID EpiFlu accession no. EPI_ISL_18607170), was sequenced from a chicken sample collected in Belgium on November 30, 2023. We used that strain as the reference strain for genotype EA-2023-DG in this study.

### Investigation of Geographic Origin of Reassortant EA-2023-DG Genotype

To retrieve a selection of the most homologous sequences to each of the EA-2023-DG viral genome segments, we conducted a search in GISAID EpiFlu on May 27, 2024. For each of the genome segments of reference strain A/*Gallus*_*gallus*/Belgium/11307_0002/2023, we identified the 500 sequences with highest nucleotide homology and downloaded the sequences of all 8 gene segments of those genomes along with any available metadata (Appendix 2). The accompanying metadata included sampling location and precise collection date. 

We used MAFFT 7.453 (*12*) to align sequences. Because genotyping identified EA-2023-DG as a reassortant containing the PB1, HA, nucleoprotein (NP), neuraminidase (NA), matrix protein (MP), and nonstructural (NS) protein segments from EA-2021-AB–like viruses, as well as PB2 and PA segments of other circulating AIVs, we assembled 3 distinct datasets. One dataset was an alignment concatenating the 6 nonrecombinant segments, PB1, HA, NP, NA, MP, and NS, of all 54 EA-2023-DG samples and the 500 most homologous H5N1 virus samples found for those 6 segments. We removed duplicates from the 6 segment searches by using a custom Python script (Python Software Foundation, https://www.python.org), resulting in a total dataset of 958 samples. The second and third datasets, 1 for PA and 1 for PB2, used a distinct PA or PB2 alignment from all 54 EA-2023-DG samples and the 500 most homologous samples for the corresponding segment. 

Because the initial concatenated alignment was computationally too large to conduct Bayesian phylogeographic inference, we conducted a preliminary maximum-likelihood phylogenetic inference in IQTREE 1.6.12 (*13*), using default settings and the best-fitting substitution model identified by ModelFinder (*14*). We used that preliminary phylogenetic inference to restrict the alignment to a large monophyletic clade of 225 samples containing the 54 EA-2023-DG samples. Thus, that phylogeny-guided downsampling reflects the evolutionary history of the larger set of 958 samples.

To assess the presence of a recombination signal within the resulting PB1-HA-NP-NA-MP-NS concatenated alignment of those 6 segments, we performed the Φ test (*15*) implemented in the SplitsTree 4.14.8 program (*16*), which confirmed the occurrence of past recombination events (p<0.001). We then used the RDP4 program (*17*) to identify recombinant samples: we identified and discarded 41 recombinants within the set of background samples, which resulted in a PB1-HA-NP-NA-MP-NS concatenated alignment of 184 samples. We performed a new Φ test on that final alignment and confirmed the absence of a remaining recombination signal.

We conducted a discrete phylogeographic analysis based on each of the 3 alignments (PA, PB2, and recombinant-free PB1-HA-NP-NA-MP-NS) to reconstruct the transition history of viral lineages among countries and investigate the country from which EA-2023-DG genotype emerged. For those analyses, the selection of countries as discrete locations was imposed by the lack of higher sampling precision for most publicly available background sequences retrieved from GISAID EpiFlu. We performed the discrete phylogeographic analyses by using the discrete diffusion model (*18*) implemented in the BEAST 1.10 software package (*19*), specifying a GTR+Γ (general time-reversible with a gamma-distributed rate heterogeneity) nucleotide substitution model (*20*), a relaxed molecular clock with an underlying log-normal distribution to model branch-specific evolutionary rates (*21*), and a skygrid coalescent model for the tree prior (*22*). We ran the PA analysis for 300 Markov chain Monte Carlo (MCMC) iterations, the PB2 analysis for 500 MCMC iterations, and the concatenated analyses for 210 million MCMC iterations and sampled posterior trees every 100,000 iterations. We assessed the MCMC convergence and mixing by using the Tracer 1.7 program (*23*), checking that all estimated parameters were associated with an effective sample size value >200. After discarding the initial 10% of sampled posterior trees as burn-in, we retrieved and annotated the maximum clade credibility (MCC) tree by using TreeAnnotator 1.10 (*19*) and eventually plotted the tree in R (The R Project for Statistical Computing, https://www.r-project.org) by using a custom script (https://github.com/sdellicour/ea-2023-dg_emergence).

#### Reconstruction of Reassortant EA-2023-DG Dissemination History

To reconstruct the spread of the EA-2023-DG genotype after the reassortment event at its genesis, we performed a continuous phylogeographic reconstruction on the basis of the concatenated alignment of all 8 genomic segments of the 54 EA-2023-DG genomes available on January 15, 2025. We then aligned sequences again using MAFFT 7.453 (*12*) and performed the Φ test (*15*) in SplitsTree (*16*) to confirm the absence of a recombination signal within the concatenated alignment made of EA-2023-DG genomic sequences. The availability of precise sampling coordinates for all considered samples in this alignment made a spatially explicit phylogeographic reconstruction possible. We performed that continuous phylogeographic reconstruction by using the relaxed random walk model (*24*,*25*) in BEAST version 1.10 (*19*). As with the discrete approach, the continuous phylogeographic approach involves a joint inference of both the phylogenetic tree representing the evolutionary relationships between sampled sequences, and the locations, in this case the geographic coordinates (latitude and longitude) of unsampled common ancestors (*26*). Specifically, we used a gamma distribution to model the among-branch heterogeneity in diffusion velocity, and modeled branch-specific evolutionary rates according to a relaxed molecular clock with an underlying log-normal distribution and the nucleotide substitution process according to a GTR+Γ parameterization. As for the tree prior, we specified a flexible skygrid model (*22*). We ran the MCMC for 250 million iterations, sampling posterior trees every 100,000 iterations, and eventually discarded the first 25 million sampled trees as burn-in. We used Tracer 1.7.2 to assess the MCMC convergence and mixing properties and ensure that estimated parameters were all associated with an effective sample size >200. We used TreeAnnotator version 1.10 to identify and annotate the MCC tree and R functions in the SERAPHIM package (*27*,*28*) to extract the spatiotemporal information embedded within the 1,000 trees sampled from the post–burn-in posterior distribution and to estimate the weighted diffusion coefficient (*29*) associated with the spread of the EA-2023-DG genotype.

## Results

### Epidemiologic Findings

**Figure 1:**
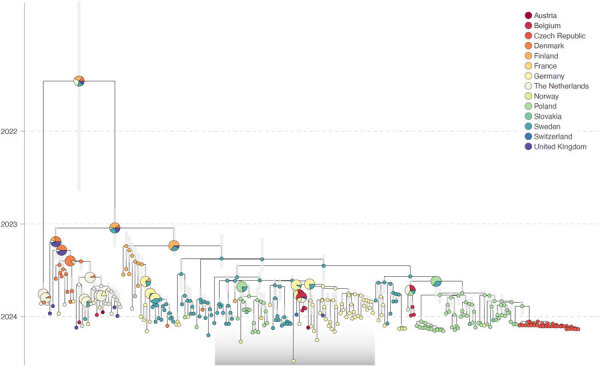
Discrete phylogeographic analysis from study of genesis and spread of novel highly pathogenic avian influenza A(H5N1) clade 2.3.4.4b virus genotype EA-2023-DG reassortant, western Europe. The maximum clade credibility (MCC) tree was obtained from discrete phylogeographic inference based on the analysis of the PB1-HA-NP-NA-MP-NS (polymerase basic 1, hemagglutinin, nucleoprotein, neuraminidase, matrix protein, nonstructural protein) concatenated alignment of EA-2023-DG samples and selected EA-2021-AB reference sequences sourced from GISAID EpiFlu (http://www.gisaid.org). Vertical gray shaded bars reflect the 95% highest posterior density interval associated with each internal node age estimate; internal nodes are colored according to their inferred location, and tip nodes are colored according to their sampling location. For the internal nodes, when a single location could not be inferred with a posterior probability >0.95, we used a pie chart to display the posterior probabilities associated with inferred locations with a posterior probability of >0.05. The gray transparent box highlights the position of the EA-2023-DG clade. The discrete phylogeographic reconstruction based on the analysis of the polymerase basic 2 and polymerase acidic segments are available as supplementary information (Appendix 1 Figures 1, 2).

Our analysis showed that the EA-2023-DG genotype was initially detected in a sample from a swan found dead on a Baltic Sea island in Finland on November 1, 2023 (GISAID accession no. EPI_ISL_19409779) (Figure 1; Appendix 1 Table 1). Then, on November 17, 2023, the EA-2023-DG genotype emerged in a hobby farm near the North Sea coast in northern Germany that had a mixed population of 52 chickens, 3 turkeys, and 11 ducks (Appendix 1 Table 1). EA-2023-DG subsequently spread to a total of 11 countries, and 54 cases were detected (Appendix 1 Table 2). The most affected countries included Germany, Sweden, and Poland. Germany was most affected, with cases in 13 poultry farms, 8 wild birds, 1 captive bird, and 1 wild mammal (red fox). Sweden experienced cases on 1 poultry farm and in 11 wild birds, and Poland had 5 poultry and 3 wild bird cases. Affected poultry were among the Phasianidae family (n = 21), which includes chickens (*Gallus gallus*) and turkeys (*Meleagris gallopavo*), and the Anatidae family (n = 1), which includes geese and ducks. Domestic poultry were only infected in Germany, Poland, Sweden, and Belgium. Captive animals included 1 captive barn owl (Tytonidae family, *Tyto alba*) and 1 black necked swan (Anatidae family, *Cygnus melancoryphus*) in a zoo. Wild birds, all found dead during passive surveillance efforts, included birds from the Anatidae (n = 24), Accipitridae (n = 3), Falconidae (n = 1), Strigidae (n = 1), Gruidae (n = 1), and Ardeidae (n = 1) families (Appendix 1 Table 1). A single mammal sample from a red fox (*Vulpes vulpes*) from the Hamburg district of Germany was EA-2023-DG positive. The most recent (June 25, 2024) occurrence was a wild goose sample in Germany.

### Genesis of AIV Genotype EA-2023-DG in Western Europe

**Figure 2:**
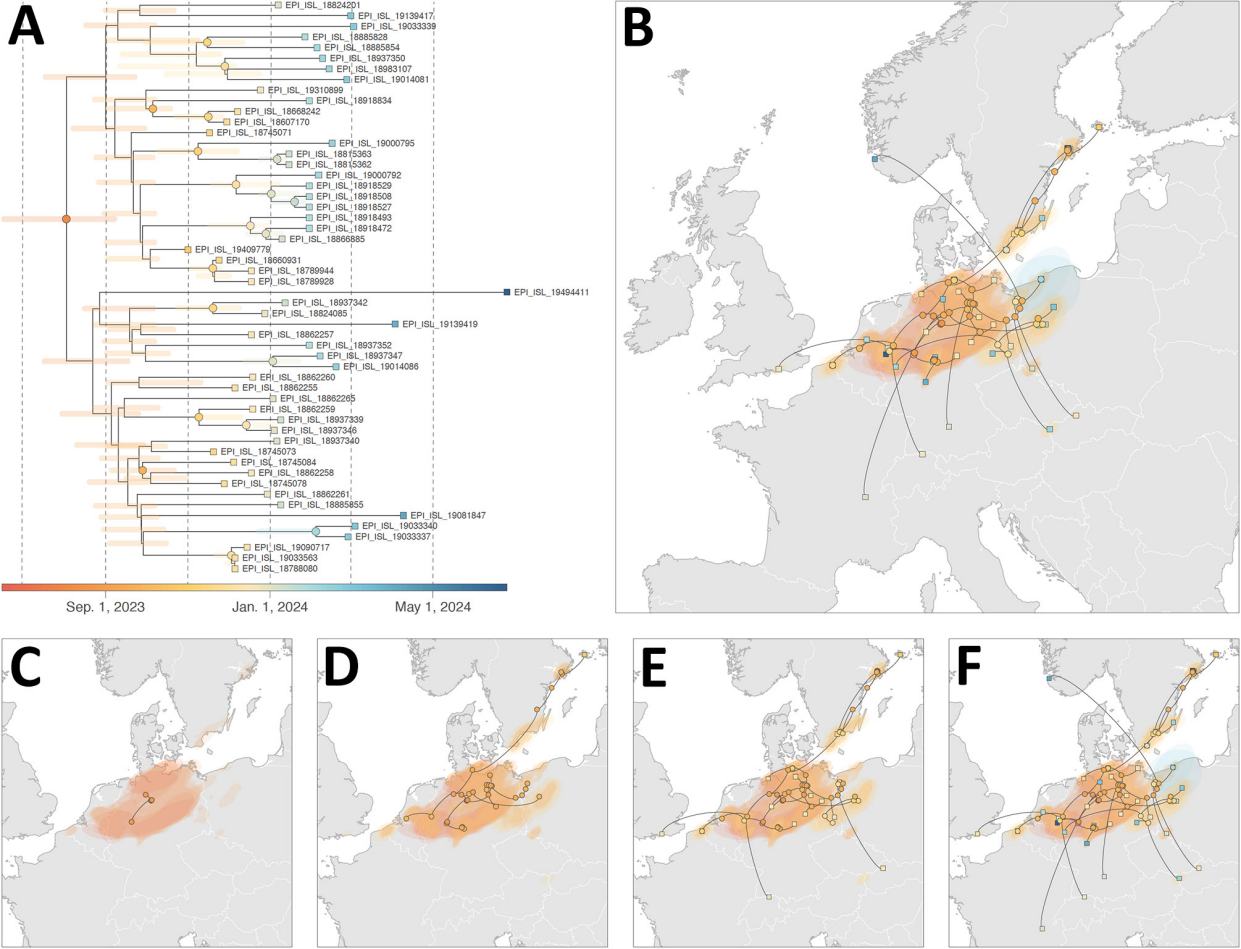
Continuous phylogeographic analysis from study of genesis and spread of novel highly pathogenic avian influenza A(H5N1) clade 2.3.4.4b virus genotype EA-2023-DG reassortant, western Europe. A) Time-scaled maximum clade credibility treeretrieved and annotated from the continuous phylogeographic inference based on analysis of 54 publicly available complete EA-2023-DG genomes and associated sampling metadata from GISAID EpiFlu (http://www.gisaid.org). The tree shows horizontal shaded bars reflecting the 95% highest posterior density (HPD) associated with each internal node age estimate. The tree and HPD estimates are based on 1,000 trees sampled from the posterior distribution of trees and are colored according to time of occurrence; internal nodes are displayed as dots and tip nodes are displayed as squares. Dotted vertical lines correspond to 2-month intervals beginning July 1, 2023. B­–F) Phylogeographic reconstruction of EA-2023-DG viral lineages across western Europe. We mapped the MCC tree and 80% HPD regions reflecting the uncertainty related to the Bayesian phylogeographic inference as of June 25, 2024 (B), and for other time points: C) September 1, 2023; D) November 1, 2023; E) January 1, 2024; and F) June 25, 2024. On the maps, the dispersal direction of viral lineages is indicated by the edge curvature in a counterclockwise direction.

The discrete phylogeographic analyses conducted on the recombinant-free PB1-HA-NP-NA-MP-NS concatenated alignment and the PB2 and PA alignments confirmed the monophyletic nature of the EA-2023-DG clade (Figure 1; Appendix 1 Figures 1, 2). The analysis based on the PB1-HA-NP-NA-MP-NS concatenated alignment inferred the origin of the EA-2023-DG clade at the end of July 2023 (July 22, 2023; 95% highest posterior density [HPD] interval June 10–August 27, 2023) in Sweden with a posterior probability >0.99 (Figure 1). The analyses of the PB2 and PA segments, inferred its origin in Germany (posterior probability = 0.99); the PB2 analysis inferred its origin on January 6, 2023 (95% HPD September 17, 2022–April 20, 2023), and the PA analysis inferred its origin on June 30, 2023 (95% HPD April 14–September 24, 2023) (Appendix 1 Figures 1, 2). 

Outcomes of discrete phylogeographic inference are known to be notably affected by sampling bias (*30*). Thus, the discrepancy between the country of origin inferred from the discrete phylogeographic analyses conducted for the recombinant-free PB1-HA-NP-NA-MP-NS concatenated alignment on the one hand and on the PB2 and PA alignments on the other could arise from the heterogeneous sampling of closely related sequences in the different countries within the study area. However, those results overall indicated that EA-2023-DG emerged during the spring and summer of 2023 in the southwestern Baltic Sea region. The continuous phylogeographic analysis further confirms the emergence of the reassortant genotype in this region and time frame (Figure 2).

### Spread History and Dynamics of EA-2023-DG

Our continuous phylogeographic analysis using concatenated EA-2023-DG sequences for all 8 segments confirmed that the ancestral node of this clade traces back to the southwestern Baltic Sea shores of Sweden, Germany, and Denmark (Figure 2, panel B). Although the most ancestral nodes of the MCC tree are inferred in northern Germany (Figure 2, panel C), the uncertainty associated with the Bayesian phylogeographic inference (shaded 80% HDP polygons) highlights a potential area of origin that is relatively large and corresponds to the larger southwestern Baltic Sea area, which aligns with the results of the discrete phylogeographic reconstructions. Our continuous phylogeographical analysis dates the most ancestral node of the EA-2023-DG clade as August 2, 2023 (95% HPD July 16–September 7, 2023), also aligning with the discrete phylogenetic analyses (Figure 2, panel A). The continuous phylogeographic analysis indicated that after its emergence, lineages of the EA-2023-DG genotype further spread in Germany and Poland, toward the Netherlands, and to southern Sweden and southern Finland before November 2023. Its lineages then reached Belgium, England, Slovakia, and Switzerland during November and December 2023. By June 2024, EA-2023-DG spread as far north as the island of Åland in south Finland; as far south as Lyon, France; as far east as Slovakia; and as far west as England (Figure 2, panels B–F). Overall, the virus predominantly circulated in Germany, Sweden, and Poland, with only occasional detections in other countries in Europe.

## Discussion

The ongoing panzootic caused predominantly by clade 2.3.4.4b HPAIVs is notorious for its diversifying evolution, including frequent reassortment events that result in an ever-changing range of circulating genotypes (*2*,*6*). Reassortment events represent crucial shifts in virus evolution that can affect host range, pathogenicity, and other epidemiologically relevant aspects of the virus phenotype; thus, understanding the dynamics behind the emergence and spread of such novel reassortants is critical. Combining complete avian influenza genomes and exact spatial and temporal sampling data enables detailed reconstruction of virus dispersal during an outbreak (*31*) and identification of reassortment events (*2*).

In this study, we analyzed all available full-genome sequences of novel reassortant HPAIV H5N1 genotype EA-2023-DG, which emerged in 2023 in western Europe (*10*), to reconstruct its genesis and dispersal dynamics. We traced its origin to the southwestern Baltic Sea area in the spring and summer of 2023. More precisely, most of the genome (i.e., PB1, HA, NP, NA, MP, and NS segments) originated from the dominant EA-2021-AB genotype, and the most recent common ancestor of those EA-2023-DG genomic segments likely emerged in or close to Sweden during summer 2023. As for the PA and PB2 segments, we inferred their origin in Germany, meaning that they could have originated from low pathogenicity avian influenza viruses circulating in Germany during winter and spring 2023, as suggested by others merely on the basis of sequence similarity (*10*). Overall, our results point toward a local reassortment event that occurred in the southwestern Baltic Sea area, which is in line with the first occurrence of the genotype in southern Finland. 

Of note, our phylogeographic reconstructions of the genesis of EA-2023-DG agree with the AIV introduction risk prediction on the basis of wild bird migration data and AIV occurrences by the EFSA Bird Flu Radar Tool (*32*). Those predictions indicated high introduction risk near the southwestern Baltic Sea along the shores of Germany, Denmark, and Sweden for the week fitting the time to most recent common ancestor (tMRCA) of the EA-2023-DG clade (August 7–13, 2023). In addition, the population density and ringing recapture data of species from the Anatidae family associated with the most distant translocations of EA-2023-DG and captured in Germany and Denmark around the week of the tMRCA according to the Migration Mapping Tool, Bird Flu Radar Tool (*32*), do not contradict the viral lineage movements we reconstructed here. Those observations confirm the continued involvement of an increasing spectrum of wild bird species in the epidemiology of HPAIV.

The spectrum of bird families affected by EA-2023-DG, including Phasianidae, Anatidae, Accipitridae, Falconidae, Strigidae, Tytonidae, Gruidae, and Ardeidae, aligns largely to the host spectrum of genotype EA-2021-AB that donated most of the genome (*2*). A single dead fox was found infected in the core area of EA-2023-DG circulation in northern Germany, confirming the role of wild carnivores as dead-end hosts of HPAIV clade 2.3.4.4b (*5*). Because of the reassortment event, we could not include ancestral DG precursor genomes in the detailed whole-genome–based phylogeographic reconstruction, resulting in a substantial spatial uncertainty covering the western Baltic Sea shores and North Sea coast of Germany. Another factor that might have contributed to the temporal and spatial uncertainty in our predictions is the lack of standardized surveillance approaches between countries, especially for wildlife surveillance. Our spatially explicit phylogeographic reconstruction highlights continued circulation with a focus in Germany, Poland, and Sweden and sporadic occurrences as far north as central Sweden, as far south as central France, as far west as England, and as far east as Slovakia.

During its period of circulation, EA-2023-DG became the second most frequent (54 cases) genotype in the countries it affected, but EA-2021-AB remained the dominant genotype with 84 reported cases. Other prevalent genotypes were EA-2023-DB (32 cases), EA-2024-DI (26 cases), EA-2022-BB (16 cases), EA-2021-I (14 cases), and EA-2023-DA (13 cases) (*33*,*34*). Ten additional genotypes circulated at lower frequency (<10 cases), reflecting the known diversification potential of H5N1 clade 2.3.4.4b viruses (*2*).

In vivo experiments following up on the emergence of HPAIV H5N1 in cattle in the United States (*35*), and its subsequent spillover to other mammals, including cats (*35*) and exposed humans (*36*), used an EA-2023-DG genotype virus as a model of contemporary circulating viruses in Europe. Those studies indicated that these viruses efficiently replicate in bovine mammary tissue and can produce adaptive mutations (PB2 E627K) during replication (*37*). Those findings underscore the value of phenotypic characterization of currently circulating H5Nx clade 2.3.4.4b viruses, including the newly emerged EA-2023-DG genotype, because the zoonotic potential of the viruses can evolve, driven and shaped by epidemiologic events that could increase the likelihood of spillover to mammals and subsequent adaptation. In response to HPAIV reassortment promiscuity resulting in fast evolution and diversification (*2*,*6*), efficient livestock and wildlife surveillance programs including a viral genomic characterization are essential. 

In conclusion, although gaps in surveillance data will always exist, we demonstrated that viral genomic data collected from surveillance programs combined with precise spatial and temporal metadata can enable a comprehensive investigation of the genesis of novel AIV reassortants and of their spread dynamics. In addition to viral genetic characterization, such as adaptive mutations and genotyping, these parameters provide vital clues for informing outbreak prevention and intervention policies.

Appendix 1 Additional information on genesis and spread of novel highly pathogenic avian influenza A(H5N1) clade 2.3.4.4b virus genotype EA-2023-DG reassortant, western Europe.

Appendix 2 Genomic data used in a study of genesis and spread of novel highly pathogenic avian influenza A(H5N1) clade 2.3.4.4b virus genotype EA-2023-DG reassortant, western Europe.
