# Supplementary material for: Genesis and Spread of Novel Highly Pathogenic Avian Influenza A(H5N1) Clade 2.3.4.4b Virus Genotype EA-2023-DG Reassortant, Western Europe
Source: Emerg Infect Dis. 2025 Jun;31(6):1100–8. doi: 10.3201/eid3106.241870 (PMC12123936; doi:10.3201/eid3106.241870)
Supplement: Appendix 1 — Additional information on genesis and spread of novel highly pathogenic avian influenza A(H5N1) clade 2.3.4.4b virus genotype EA-2023-DG reassortant, western Europe. [file 24-1870-Techapp-s1.pdf]

Article DOI: https://doi.org/10.3201/eid3106.241870

EID cannot ensure accessibility for supplementary materials supplied by authors. Readers who have difficulty accessing supplementary content should contact the authors for assistance.

Genesis and Spread of Novel Highly Pathogenic Avian Influenza A(H5N1) Clade 2.3.4.4b Virus Genotype EA-2023-DG Reassortant, Western Europe

Appendix 1

Appendix 1 Table 1. Accession numbers and metadata of all EA-2023-DG genomes included in the study

Isolate_ID	Isolate name	Country	Host	Collection date	Animal specimen source	Animal health status	Domestic status	Family
EPI_ISL_18607170	A/Gallus_gallus/Belgium/11307_0002/2023	Belgium	*Gallus gallus*	2023–11–30	Oropharyngeal-cloacal swab	Dead	Domestic	Phasianidae
EPI_ISL_18660931	A/barnacle_goose/Sweden/SVA231201SZ0276/FB004873/K-2023	Sweden	*Branta leucopsis*	2023–11–24	Oro-pharyngeal swab	Dead	Wild	Anatidae
EPI_ISL_18668242	A/Gallus_gallus/Belgium/11632_0003/2023	Belgium	*Gallus gallus*	2023–12–08	Oropharyngeal-cloacal swab	Dead	Domestic	Phasianidae
EPI_ISL_18745071	A/chicken/Germany-NI/2023AI08838/2023	Germany	*Gallus gallus*	2023–11–17	Oropharyngeal-cloacal swab	Dead	Domestic	Phasianidae
EPI_ISL_18745073	A/turkey/Germany-MV/2023AI08844/2023	Germany	*Meleagris gallopavo*	2023–11–20	Oropharyngeal-cloacal swab	Dead	Domestic	Phasianidae
EPI_ISL_18745078	A/turkey/Germany-BB/2023AI09073/2023	Germany	*Meleagris gallopavo*	2023–11–28	Swab	Dead	Domestic	Phasianidae
EPI_ISL_18745084	A/domestic_goose/Germany-MV/2023AI09159/2023	Germany	*Anatidae*	2023–12–05	Swab	Dead	Domestic	Anatidae
EPI_ISL_18788080	A/turkey/Poland/H542-NM/2023	Poland	*Meleagris gallopavo*	2023–12–06	Organ (liver, lungs, brain, spleen, intestines)	Dead	Domestic	Phasianidae
EPI_ISL_18789928	A/barnacle_goose/Sweden/SVA231221SZ0336_/FB005040/K-2023	Sweden	*Branta leucopsis*	2023–12–18	Oro-pharyngeal swab	Dead	Wild	Anatidae
EPI_ISL_18789944	A/barnacle_goose/Sweden/SVA231221SZ0336_/FB005041/K-2023	Sweden	*Branta leucopsis*	2023–12–18	Oro-pharyngeal swab	Dead	Wild	Anatidae
EPI_ISL_18815362	A/Turkey/Sweden/SVA240116SZ0719/FB010937/M-2024	Sweden	*Meleagris gallopavo*	2024–01–15	Oro-pharyngeal swab	Dead	Domestic	Phasianidae
EPI_ISL_18815363	A/Turkey/Sweden/SVA240116SZ0719/FB010938/M-2024	Sweden	*Meleagris gallopavo*	2024–01–15	Oro-pharyngeal swab	Dead	Domestic	Phasianidae

Isolate_ID	Isolate name	Country	Host	Collection date	Animal specimen source	Animal health status	Domestic status	Family
EPI_ISL_18824085	A/Canada_Goose/England/142157/2023	United Kingdom	*Branta canadensis*	2023–12–28	Oro-pharyngeal swab and cloacal swab	Dead	Wild	Anatidae
EPI_ISL_18824201	A/greylag_goose/France/24P000660/2024	France	*Greylag goose*	2024–01–07	Oro-pharyngeal swab	Dead	Wild	Anatidae
EPI_ISL_18862255	A/Bean_goose/Germany-BE/2023AI09307/2023	Germany	*Anser fabalis*	2023–12–06	Organ	Dead	Wild	Anatidae
EPI_ISL_18862257	A/Common_Crane/Germany-TH/2023AI09495/2023	Germany	*Grus grus*	2023–12–18	Organ	Dead	Wild	Gruidae
EPI_ISL_18862258	A/Common_Kestrel/Germany-NW/2023AI09478/2023	Germany	*Falco tinnunculus*	2023–12–18	Oropharyngeal-cloacal swab	Dead	Wild	Falconidae
EPI_ISL_18862259	A/chicken/Germany-SH/2023AI09451/2023	Germany	*Gallus gallus*	2023–12–19	Oropharyngeal-cloacal swab	Dead	Domestic	Phasianidae
EPI_ISL_18862260	A/chicken/Germany-ST/2023AI09470/2023	Germany	*Gallus gallus*	2023–12–19	Organ	Dead	Domestic	Phasianidae
EPI_ISL_18862261	A/Black-necked_Swan/Germany-BB/2023AI09499/2023	Germany	*Cygnus melancoryphus*	2023–12–30	Organ	Dead	Captive	Anatidae
EPI_ISL_18862265	A/chicken/Germany-MV/2024AI00101/2024	Germany	*Gallus gallus*	2024–01–03	Oropharyngeal-cloacal swab	Dead	Domestic	Phasianidae
EPI_ISL_18866885	A/mallard/Sweden/SVA240117SZ0347/FB000141/AB-2024	Sweden	*Mallard*	2024–01–10	Oro-pharyngeal swab	Dead	Wild	Anatidae
EPI_ISL_18885828	A/chicken/Poland/H22-T2/2024	Poland	*Gallus gallus*	2024–01–27	Oro-pharyngeal swab	Sick	Domestic	Phasianidae
EPI_ISL_18885854	A/turkey/Poland/H36-T3/2024	Poland	*Meleagris gallopavo*	2024–02–05	Oro-pharyngeal swab	Sick	Domestic	Phasianidae
EPI_ISL_18885855	A/mute_swan/Poland/MB021-N/2024	Poland	*Cygnus olor*	2024–01–09	Organ (liver, lungs, brain, intestines)	Dead	Wild	Anatidae
EPI_ISL_18918472	A/Muteswan/Sweden/SVA240202SZ0099/FB000320/AB-2024	Sweden	*Cygnus olor*	2024–01–30	Oro-pharyngeal swab	Sick	Wild	Anatidae
EPI_ISL_18918493	A/Muteswan/Sweden/SVA240202SZ128/FB000337/AB-2024	Sweden	*Cygnus olor*	2024–01–30	Oro-pharyngeal swab	Sick	Wild	Anatidae
EPI_ISL_18918508	A/Muteswan/Sweden/SVA240202SZ128/FB000338/AB-2024	Sweden	*Cygnus olor*	2024–01–30	Oro-pharyngeal swab	Sick	Wild	Anatidae
EPI_ISL_18918527	A/Muteswan/Sweden/SVA240202SZ128/FB000344/AB-2024	Sweden	*Cygnus olor*	2024–01–30	Oro-pharyngeal swab	Sick	Wild	Anatidae
EPI_ISL_18918529	A/Muteswan/Sweden/SVA240202SZ128/FB000345/AB-2024	Sweden	*Cygnus olor*	2024–01–30	Oro-pharyngeal swab	Sick	Wild	Anatidae
EPI_ISL_18937339	A/chicken/Germany-SH/2024AI00169/2024	Germany	*Gallus gallus domesticus*	2024–01–09	Swab	Dead	Domestic	Phasianidae
EPI_ISL_18937340	A/turkey/Germany-BY/2024AI00164/2024	Germany	*Meleagris gallopavo*	2024–01–06	Oropharyngeal-cloacal swab	Dead	Domestic	Phasianidae
EPI_ISL_18937342	A/wild_goose/Germany-NW/2024AI00581/2024	Germany	*Anatidae*	2024–01–12	Oropharyngeal-cloacal swab	Dead	Wild	Anatidae
EPI_ISL_18937346	A/chicken/Germany-SH/2024AI00133/2024	Germany	*Gallus gallus*	2024–01–04	Oropharyngeal-cloacal swab	Dead	Domestic	Phasianidae
EPI_ISL_18937347	A/egret/Germany-HE/2024AI01153/2024	Germany	*Ardeidae*	2024–02–07	Oropharyngeal-cloacal swab	Dead	Wild	Ardeidae
EPI_ISL_18937350	A/Turkey/Germany-NW/2024AI01054/2024	Germany	*Meleagris gallopavo*	2024–02–09	Oropharyngeal-cloacal swab	Dead	Domestic	Phasianidae
EPI_ISL_18937352	A/chicken/Germany-HE/2024AI00792/2024	Germany	*Gallus gallus*	2024–01–30	Cloacal swab	Dead	Domestic	Phasianidae

Isolate_ID	Isolate name	Country	Host	Collection date	Animal specimen source	Animal health status	Domestic status	Family
EPI_ISL_19000792	A/Canadagoose/Sweden/SVA240212SZ0129/FB000502/AB-2024	Sweden	*Branta canadensis*	2024–02–06	Oro-pharyngeal swab	Dead	Wild	Anatidae
EPI_ISL_19000795	A/Muteswan/Sweden/SVA240221SZ0306/FB000690/H-2024	Sweden	*Cygnus olor*	2024–02–16	Oro-pharyngeal swab	Dead	Wild	Anatidae
EPI_ISL_19014081	A/chicken/Germany-SN/2024AI01300/2024	Germany	*Gallus gallus*	2024–02–27	Oropharyngeal-cloacal swab	Dead	Domestic	Phasianidae
EPI_ISL_19014086	A/swan/Germany-HE/2024AI01282/2024	Germany	*Cygnus*	2024–02–19	Oropharyngeal-cloacal swab	Sick	Wild	Anatidae
EPI_ISL_19033337	A/chicken/Poland/H79-T2/2024	Poland	*Gallus gallus*	2024–02–28	Oro-pharyngeal swab	Sick	Domestic	Phasianidae
EPI_ISL_19033339	A/buzzard/Poland/MB098-N/2024	Poland	*Buteo buteo*	2024–03–03	Organ (liver, lungs, brain, spleen, intestines)	Dead	Wild	Accipitridae
EPI_ISL_19033340	A/buzzard/Poland/MB103-N/2024	Poland	*Buteo buteo*	2024–03–04	Organ (liver, lungs, brain, spleen, intestines)	Dead	Wild	Accipitridae
EPI_ISL_19033563	A/turkey/Poland/H543-NM/2023	Poland	*Meleagris gallopavo*	2023–12–06	Organ (liver, lungs, brain, spleen, intestines)	Dead	Domestic	Phasianidae
EPI_ISL_19090717	A/Tyto_alba/Slovakia/Vh-2014/2023	Slovakia	*Tyto alba*	2023–12–15	Oro-pharyngeal swab	Dead	Captive	Tytonidae
EPI_ISL_19310899	A/Cygnus_olor/Kleinandelfingen/V0969/2023	Switzerland	*Cygnus Olor*	2023–12–25	Oropharyngeal-cloacal swab	Dead	Wild	Anatidae
EPI_ISL_19081847	A/Buzzard/Norway/2024–07–136/2024	Norway	*Buteo buteo*	2024–04–09	Tracheal swab	Dead	Wild	Accipitridae
EPI_ISL_18983107	A/owl/Austria/24020512–001/2024	Austria	*Strigidae*	2024–02–14	Pooled organs (brain, lung, trachea, liver, pancreas)	Dead	Wild	Strigidae
EPI_ISL_18918834	A/Mallard/Netherlands/24001874–001/2024	Netherlands	*Anas platyrhynchos*	2024–01–30	Cloacal swab	Dead	Wild	Anatidae
EPI_ISL_19139417	A/fox/Germany-HH/2024AI01565/2024	Germany	*Vulpes vulpes*	2024–03–01	Organ	Dead	Wild	Canidae
EPI_ISL_19139419	A/Greylag_Goose/Germany-HE/2024AI01561/2024	Germany	*Anser anser*	2024–04–03	Oropharyngeal-cloacal swab	Dead	Wild	Anatidae
EPI_ISL_19494411	A/wild_goose/Germany-NW/2024AI02730/2024	Germany	*wild goose*	2024–06–25	Oropharyngeal-cloacal swab	Dead	Wild	Anatidae
EPI_ISL_19409779	A/whooper_swan/Finland/13471/2023	Finland	*Cygnus cygnus*	2023–11–01	Tracheal swab	Dead	Wild	Anatidae

Appendix 1 Table 2. Distribution per country of poultry and wildlife cases affected by AIV genotype EA-2023-DG, November 1, 2023–June 25, 2025

Country	Poultry	Captive birds	Wild animals
Germany	13	1	8 birds and 1 fox
Sweden	2 (1 farm)	0	11
Poland	5	0	3
Belgium	2	0	0
France	0	0	1
United Kingdom	0	0	1
Slovakia	0	1	0
Norway	0	0	1
Austria	0	0	1
Switzerland	0	0	1
The Netherlands	0	0	1
Finland	0	0	1

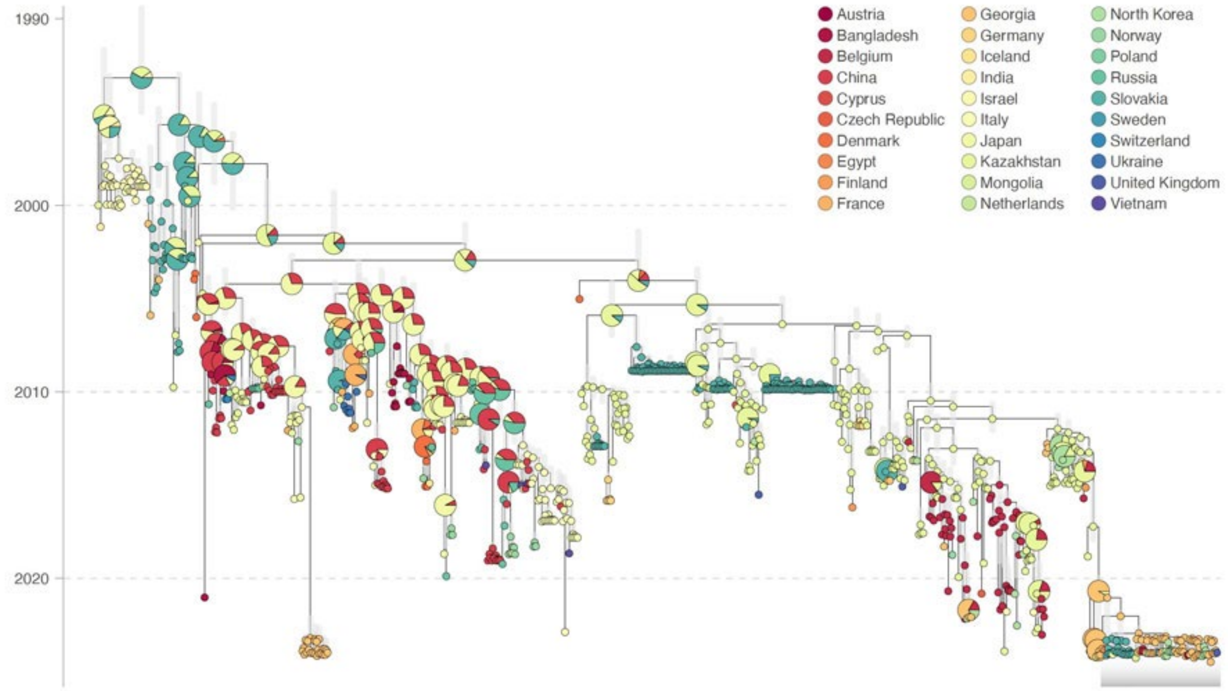

Appendix 1 Figure 1. Discrete phylogeographic analysis of the emergence of AIV H5N1 genotype EA-2023-DG, here based on sequences of the PB2 segment. We here report the time-scaled maximum clade credibility (MCC) tree obtained from the discrete phylogeographic inference based on the PB2 alignment, with vertical transparent gray line segments reflecting the 95% highest posterior density (HPD) associated with each internal node age estimate, as well as internal and tip nodes colored according to the inferred sampling and location, respectively. For the internal nodes, when there is not a single location inferred with a posterior probability >0.95, we use a pie chart to display the posterior probabilities associated with inferred locations with at least a posterior probability >0.05. The gray transparent boxplot highlights the position of the EA-2023-DG clade. See Appendix 1 Figures 1 and 2 for the discrete phylogeographic reconstruction based on the PB1-HA-NP-NA-MP-NS concatenated and PA alignments, respectively.

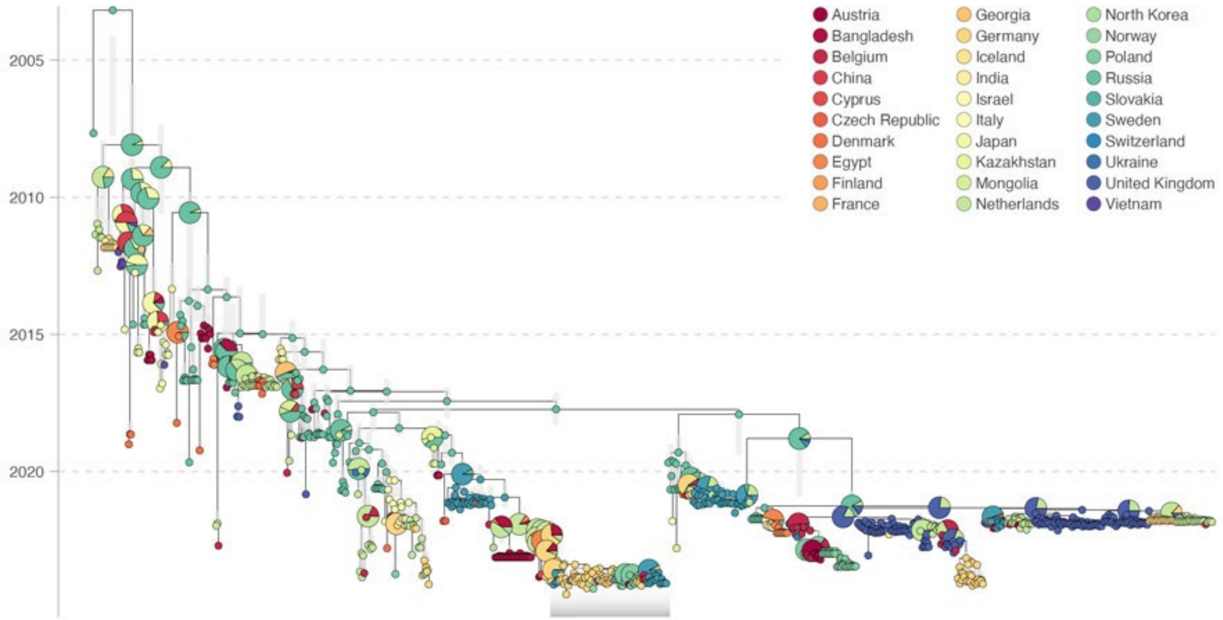

Appendix 1 Figure 2. Discrete phylogeographic analysis of the emergence of AIV H5N1 genotype EA-2023-DG, here based on sequences of the PA segment. We here report the time-scaled maximum clade credibility (MCC) tree obtained from the discrete phylogeographic inference based on the PA alignment, with vertical transparent gray line segments reflecting the 95% highest posterior density (HPD) associated with each internal node age estimate, as well as internal and tip nodes colored according to the inferred sampling and location, respectively. For the internal nodes, when there is not a single location inferred with a posterior probability >0.95, we use a pie chart to display the posterior probabilities associated with inferred locations with at least a posterior probability >0.05. The gray transparent boxplot highlights the position of the EA-2023-DG clade. See Figures 1, Appendix 1 Figure 1 for the discrete phylogeographic reconstruction based on the PB1-HA-NP-NA-MP-NS concatenated and PB2 alignments, respectively.
